# A novel difficulty scoring system of laparoscopic liver resection for liver tumor

**DOI:** 10.3389/fonc.2022.1019763

**Published:** 2022-09-29

**Authors:** Cheng Xi, Maoqun Zhu, Tianhao Ji, Yulin Tan, Lin Zhuang, Zhiping Yuan, Zheng Zhang, Litian Xu, Zhilin Liu, Xuezhong Xu, Wenbo Xue, Wei Ding

**Affiliations:** ^1^ Department of General Surgery, Wujin Hospital Affiliated with Jiangsu University, Changzhou, China; ^2^ Department of General Surgery, The Wujin Clinical college of Xuzhou Medical University, Changzhou, China; ^3^ Department of Hepatological Surgery, Affiliated Hospital of Jiangnan University, Wuxi, China; ^4^ Department of General Surgery, The First Affiliated Hospital of Nanjing Medical University, Nanjing, China; ^5^ Department of Digestion, Wujin Hospital Affiliated with Jiangsu University, Changzhou, China; ^6^ Department of Gastrointestinal Surgery, The Fifth General Hospital of Kunming, Kunming, China; ^7^ Department of Gastrointestinal Surgery, The Third Affiliated Hospital of Soochow University, Changzhou, China; ^8^ Changzhou Key Laboratory of Molecular Diagnostics and Precision Cancer Medicine, Wujin Hospital Affiliated with Jiangsu University, Changzhou, China

**Keywords:** difficulty scoring system, laparoscopic surgery, liver resection, liver tumor, outcome

## Abstract

**Objectives:**

To develop a novel difficulty scoring system (NDSS) to predict the surgical difficulty of laparoscopic hepatectomy.

**Patients and methods:**

A total of 138 patients with liver tumors performed liver resection (LLR) between March 2017 to June 2022 were selected from Affiliated Hospital of Jiangnan University and Wujin Hospital Affiliated with Jiangsu University.

Patient demographics, laboratory tests, intraoperative variables, pathological characteristics were assessed. We also assessed the Child Pugh score and the DSS-B score.

**Results:**

Patients were divided into training and testing cohort according to their hospital. Patients in training cohort were divided into high and low difficult groups based on operation time, blood loss and conversion. Higher percentage of patients with malignant liver tumor (87.0% vs. 58.1%; *P* = 0.003) or history of hepatobiliary surgery (24.1% vs. 7.0%; *P* = 0.043) in high difficult group than in low difficult group. To improve the difficulty scoring system, we incorporated the history of hepatobiliary surgery and nature of the tumor. A novel difficulty scoring system was established. The results showed that the operation time (*P* < 0.001), blood loss (*P* < 0.001), ALT (*P* < 0.001) and AST (*P* = 0.001) were associated with the novel difficulty score significantly. Compared with DSS-B, the NDSS has a higher area under the receiver operating characteristic (AUROC) (0.838 vs. 0.814). The nomogram was established according to the NDSS. The AUROCs of the nomogram in training and testing cohort were 0.833 and 0.767. The calibration curves for the probability of adverse event showed optimal agreement between the probability as predicted by the nomogram and the actual probability.

**Conclusions:**

We developed a nomogram with the NDSS that can predict the difficulty of LLR. This system could more accurately reflect the difficulty of surgery and help liver surgeons to make the surgical plan and ensure the safety of the operation.

## Introduction

With the first case of laparoscopic liver resection (LLR) reported in 1992, LLR as a treatment for liver tumors has been developed in major centres (1, 2). In the early days after LLR was introduced, it was limited to local hepatectomy, but now expanded hemihepatectomy and laparoscopic repeat liver resection (LRLR) are no longer contraindicated (3). Compared to open liver resection (OLR), there was less blood loss, shorter hospital stays, and fewer postoperative complications (4). In 2008, the first International Consensus Conference on Laparoscopic Hepatectomy (ICCLLR) was held in the United States, where LLR was identified as a safe and effective treatment for liver disease (5). And in 2014, the second ICCLLR was held in Japan, where the surgical indications were expanded and highlighted the assessment of surgical difficulty was believed important (6). The most used difficulty scoring system was Ban Difficulty Scoring System (DSS-B), which was developed by Japanese scientists Ban in 2014 (7). The scoring system included five factors: the extent of liver resection, tumor location, tumor size, proximity to major blood vessels, and Child-Pugh score of liver function. With the development of LLR around the world in recent years, some other factors affecting the difficulty of LLR have been gradually found. For instance, Uchida et al. assessed the surgical outcomes of LLR in patients with liver cirrhosis with specific reference to a difficulty scoring system (8). Kinoshita et al. investigate the predictive factors and classifications for the difficulty of laparoscopic repeated liver resection (LRLR) in patients with recurrent hepatocellular carcinoma (9). In addition, Takase et al. found that the operation time of LRLR was longer than that of laparoscopic primary liver resection (LPLR). Moreover, there was no score for caudate lobe tumors in DSS-B. Based on the above, we believed that some other factors including the history of hepatectomy may also increase the difficulty of LLR. Therefore, we intend to develop a novel difficulty scoring system (NDSS) to predict the surgical difficulty of laparoscopic hepatectomy.

## Methods

### Patients

From December 2020 to March 2022, 97 patients (training cohort) who performed LLR for liver tumor were selected at the Department of Hepatological Surgery, Affiliated Hospital of Jiangnan University. From March 2017 to June 2022, 41 patients (testing cohort) who performed LLR for liver tumor were selected at the Department of General Surgery, Wujin Hospital Affiliated with Jiangsu University. Patients who had also undergone lymph node dissection or other organ resection (except cholecystectomy) were excluded. This research was approved by the Ethics Committee of the Affiliated Hospital of Jiangnan University (LS2021078) and Wujin Hospital Affiliated with Jiangsu University (2022-SR-084).

### Data collection

**Figure 1:**
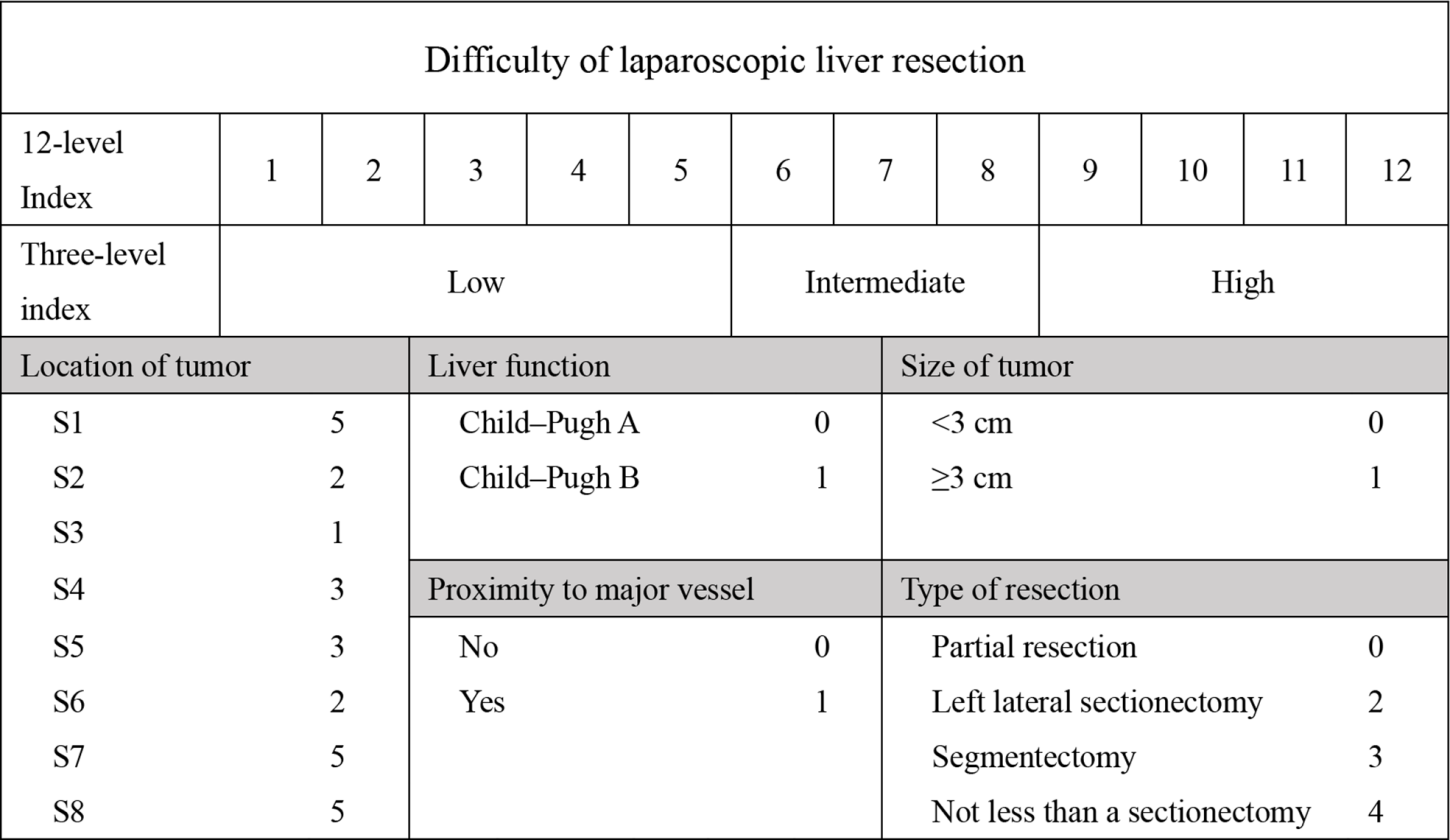
Indexes of difficulty of laparoscopic liver resection based on LLR-B.

Patient demographics included age, gender, comorbidity, and history of surgery. Laboratory tests included alanine aminotransferase (ALT), aspartate aminotransferase (AST), albumin (ALB), prothrombin time (PT), total bilirubin (TB), white blood cell count (WBC), C-reactive protein (CRP). Intraoperative variables included operation time, blood loss, blood transfusion and postoperative stay (POS). Pathological characteristics included tumor size, tumor position, and pathological pattern. We also assessed the Child-Pugh score and the DSS-B score (7, 10). To accommodate all patients, we rated caudate lobe tumors at 5 points. The detailed grading is shown in **Figure 1**. Postoperative complications included haemorrhage, bile leakage, ileus, pneumonia, pleural effusion, abdominal infection, liver failure, and incision infection. Postoperative hospitalization days were also recorded.

### Statistical analysis

Statistical analyses were conducted with Prism 9.0.1 (GraphPad Software, LLC). For continuous variables, data were expressed as mean ± standard deviation (SD), and the differences between the two groups were analyzed by the two independent samples Student t-test and Mann Whitney test. The differences among groups (more than two) were analyzed by one-factor analysis of variance (One-Way ANOVA). For categorical variables, the differences between groups were analyzed by the chi-square test, Chi-square with Yates’ correction, and Fisher’s exact test according to the sample size. Linear regression was used to predict the correlation between variables. The accuracy of different difficulty scoring systems was compared by the receiver operating characteristic (ROC) curve. Calibration plot for incidence of high difficulty was generated to assess the performance characteristics of the constructed difficulty scoring systems. The nomograms were established by the “rms” package in R version 4.2.0. We also draw the calibration plots for the adverse event rate were generated to assess the performance characteristics of the constructed nomograms. Bootstraps with 1000 resample were used for validation of the nomogram and C-index. The ROC curve and calibration plot were drawn by RStudio software (Version 1.4.1103).

## Results

### Patient characteristics and surgical outcomes

**Figure 2:**
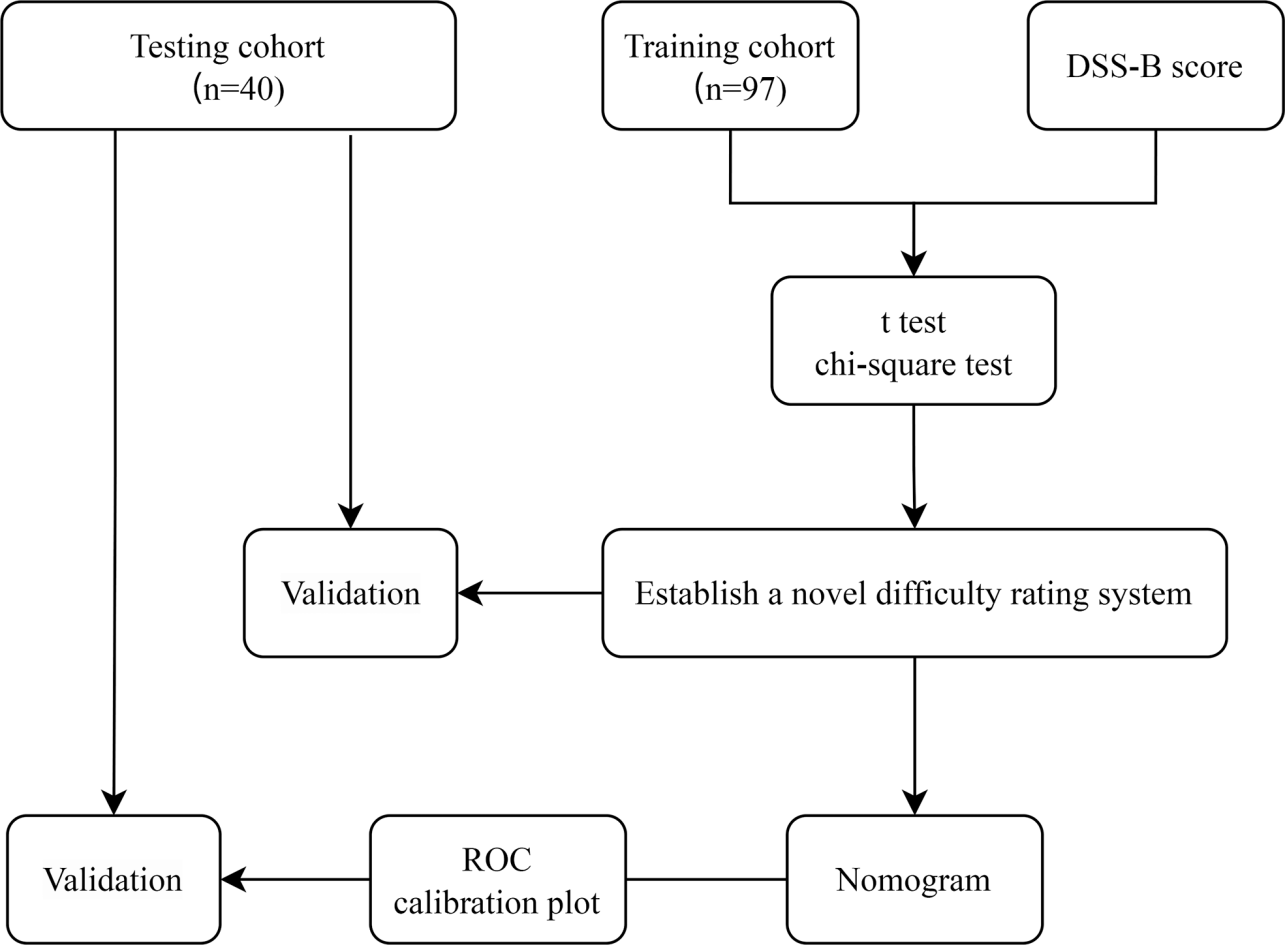
The flowchart of our research. ROC, receiver operating characteristic.

The flow chart of the study is shown in **Figure 2**. Among the 97 patients in training cohort, 40 (41.2%) patients had a history of abdominal surgery and 16 (16.5%) patients had a history of hepatobiliary surgery in them. Based on the preoperative history of hepatobiliary surgery, we classified patients into two groups: laparoscopic liver resection after previous hepatobiliary surgery (LLRAH) and laparoscopic liver resection with no hepatobiliary surgery (LLRNH). The characteristics and surgical outcomes of LLR between LLRAH and LLRNH groups were shown in **Table 1**. The results showed that patients in the LLRAH group were older and had more comorbidities compared to those in the LLRNH group (65.1 ± 8.6 vs. 56.2 ± 12.7; *P* = 0.009). And the operation time of LLR for patients in the LLRAH group was longer than that in the LLRNH group. Therefore, we believed that the history of hepatobiliary surgery was one of the important factors affecting the difficulty of LLR.

**Table 1:** Patient characteristics and surgical outcomes of LLR between LLRAH and LLRNH.

Characteristic	LLRAH (16)	LLRNH (81)	*P* value
Age, years	65.1 ± 8.6	56.2 ± 12.7	0.009^*^
Gender, male/female	11/5	50/31	0.595
Hypertension, yes/no	10/6	21/60	0.004^*^
Diabetes, yes/no	7/9	10/71	0.003^*^
POS, days	10.6 ± 4.0	11.3 ± 6.8	0.722^#^
Child-Pugh, A/B	15/1	80/1	0.743^$^
Tumor size, mm	38.5 ± 29.9	49.1 ± 29.5	0.195
DSS-B, L/I/H^a^	3/6/7	23/34/24	0.256
Pringle, yes/no	13/3	69/12	0.984^$^
Operation time, min	291.6 ± 90.9	221.3 ± 99.8	0.011^*^
Bleeding, ml	343.8 ± 392.0	258.2 ± 307.6	0.508
Transfusion, yes/no	2/14	11/70	0.775^$^
Conversion, yes/no	1/15	7/74	0.858^$^
Malignant, yes/no	15/1	57/24	0.101^$^
Postoperative morbidity	3/13	13/68	0.918^$^
Hemorrhage, yes/no	0/16	1/80	n.s^†^
Bile leakage, yes/no	0/16	4/77	n.s^†^
Ileus, yes/no	0/16	1/80	n.s^†^
Pneumonia, yes/no	1/15	2/79	0.994^$^
Pleural effusion, yes/no	1/15	2/79	0.994^$^
Abdominal infection, yes/no	1/15	1/80	0.743^$^
Liver failure, yes/no	0/16	1/80	n.s^†^
Incision infection, yes/no	0/16	2/80	n.s^†^
Postoperative day 1 (POD1)			
ALT, U/L	249.6 ± 209.4	445.8 ± 501.0	0.170^#^
AST, U/L	263.3 + 186.8	401.4 + 390.2	0.363^#^
TB, umol/L	19.1 ± 9.3	22.0 ± 14.1	0.431
WBC, *10^9/L	11.5 ± 4.3	12.3 ± 4.2	0.487
CRP, mg/L	44.1 ± 36.2	42.6 ± 40.5	0.889

LLR, laparoscopic liver resection; LLRAH, laparoscopic liver resection after previous hepatobiliary surgery; LLRNH, laparoscopic liver resection with no hepatobiliary surgery; POS, postoperative stay; DSS-B, Ban Difficulty Scoring System; n.s, not significant; ALT, alanine aminotransferase; AST, aspartate aminotransferase; TB, total bilirubin; WBC, white blood cell count; CRP, C-reactive protein.

^*^Statistically significant; ^#^Mann-Whitney test; ^$^Chi-square with Yates’ correction, ^†^Fisher’s exact test.

a L, low (1–5); I, intermediate (6–8); H, high (9–12).

### Establish and validate a novel difficulty rating system

**Figure 3:**
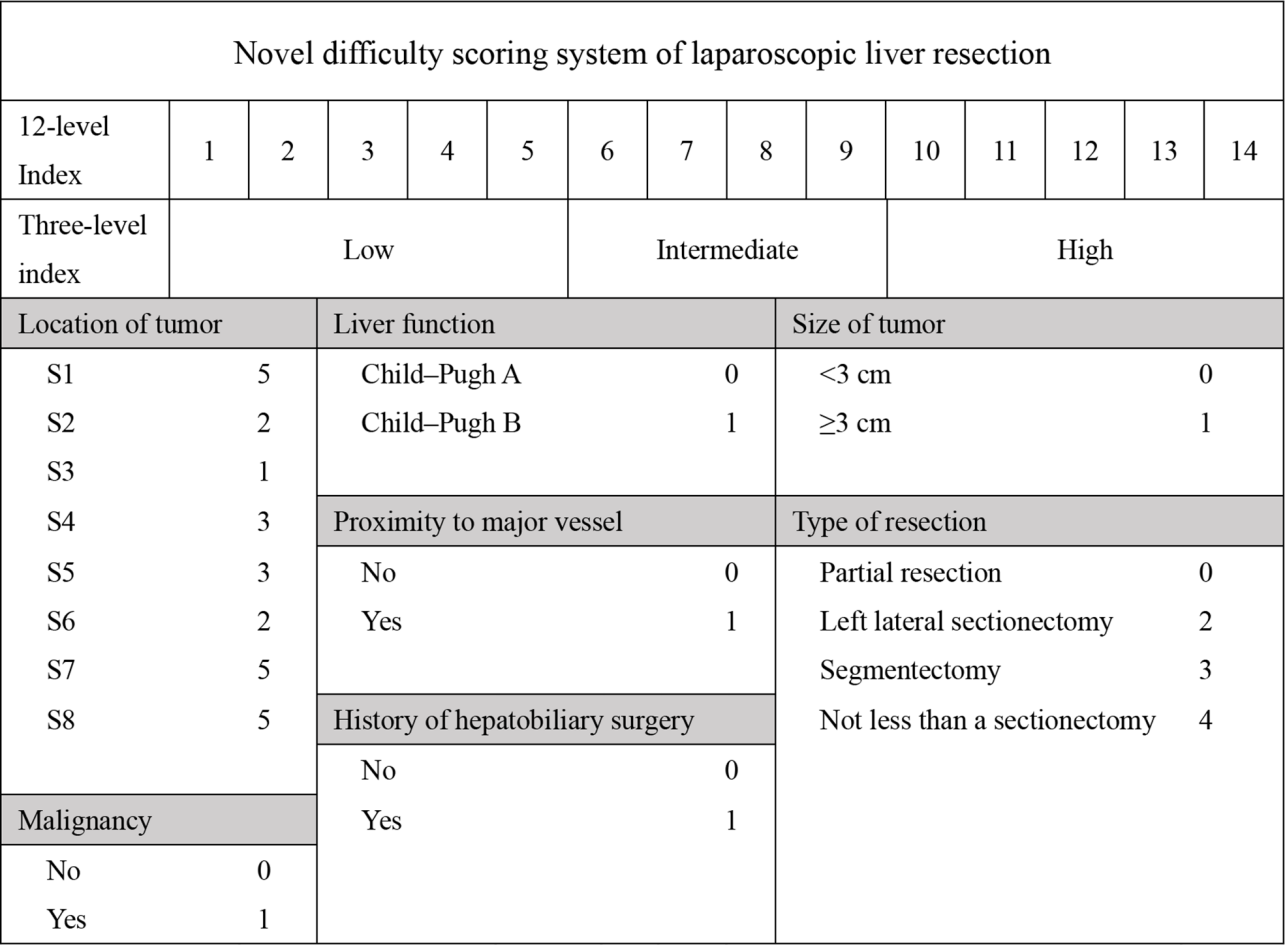
Indexes of the novel difficulty of laparoscopic liver resection.

**Figure 4:**
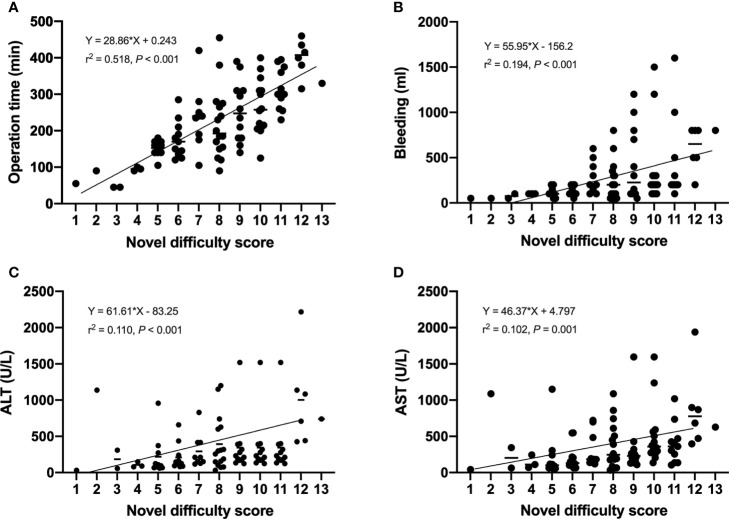
Operation time **(A)**, bleeding **(B)**, ALT **(C)**, and AST **(D)** according to difficulty score.

**Figure 5:**
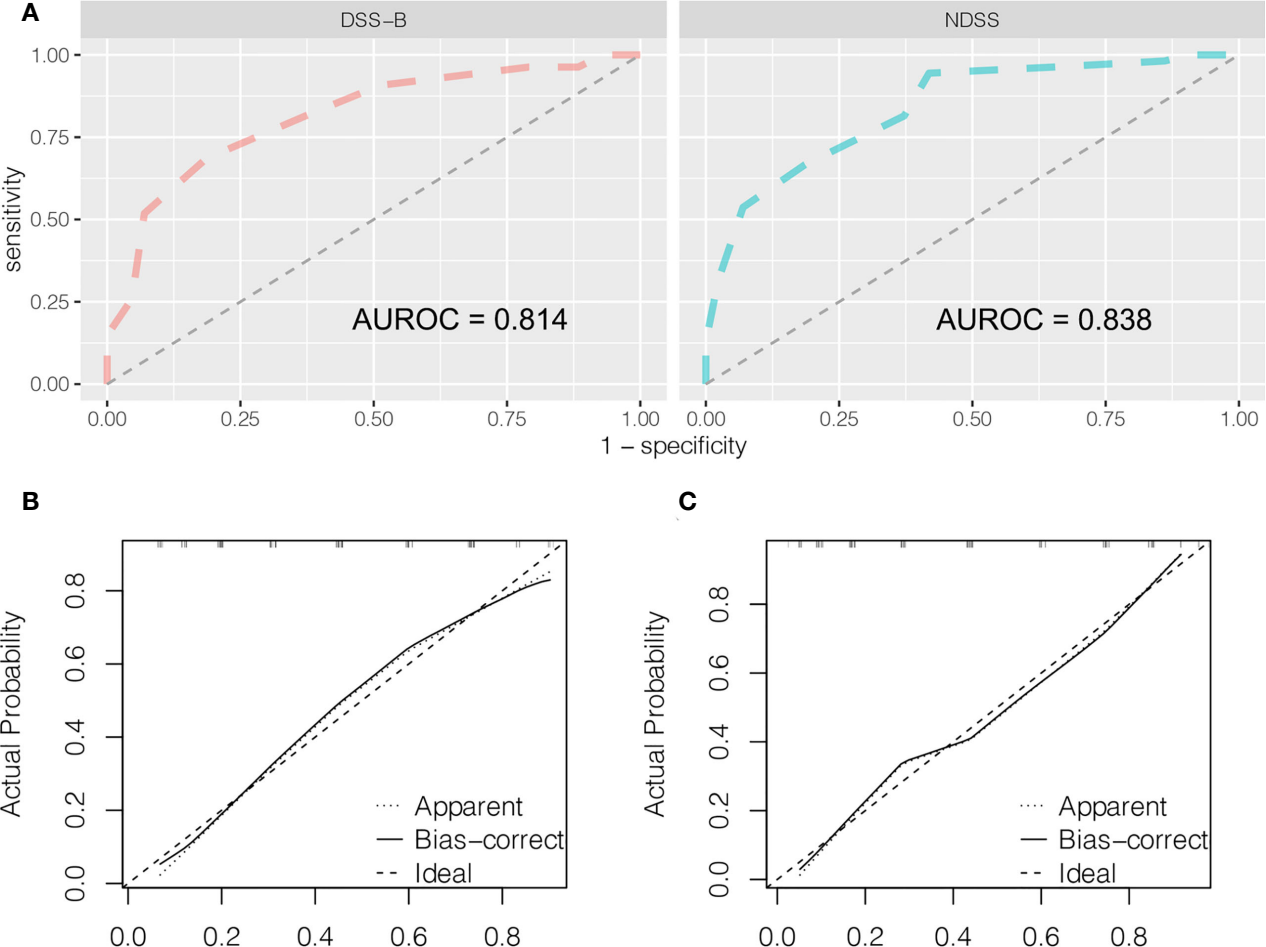
Predictive accuracy comparison of DSS-B and NDSS by ROC curve analyses **(A)**. The calibration curves for predicting surgical difficulty by DSS-B **(B)** and NDSS **(C)**.

It is well known that the operation time, blood loss and conversion to open surgery reflect surgical difficulty. An adverse event was defined when the operation time exceeded 240 minutes or the blood loss exceeded 400 ml, or when the operation was switched to open surgery. Therefore, we believed that the operation was difficult when adverse event occurred. Based on this, patients were divided into high and low difficult group. The characteristics and surgical outcomes of LLR between high difficult and low difficult groups were shown in **Table 2**. The results showed that patients in high difficult group were older (60.1 ± 11.4 vs. 54.5 ± 13.3; *P* = 0.030) and had higher difficult score (DSS-B, high difficult, 51.9% vs. 7.0%, P < 0.001) compared to those in low difficult group. And higher percentage of patients with malignant liver tumor (87.0% vs. 58.1%; *P* = 0.003) or history of hepatobiliary surgery (24.1% vs. 7.0%; *P* = 0.043) in high difficult group than in low difficult group. To improve the difficulty scoring system, we incorporated the history of hepatobiliary surgery and the nature of the tumor. A novel difficulty scoring system was established as shown in **Figure 3**. A history of hepatobiliary surgery or malignancy was each assigned a score of 1. The NDSS for a liver tumor in this study ranged from 1 to 13 (**Table 3**; **Figure 4**). The results showed that the operation time (*P* < 0.001), blood loss (*P* < 0.001), transfusion (*P* < 0.001), ALT (*P* < 0.001) and AST (*P* = 0.001) were associated with the novel difficulty score significantly. The area under the receiver operating characteristic (AUROC) was used to verify the accuracy of the NDSS in predicting surgical difficulty of LLR for patients with a liver tumor. Compared with DSS-B, the NDSS has a higher AUROC (0.838 vs. 0.814, **Figure 5A**). The C-index of the DSS-B was 0.814 (95% CI: 0.731-0.897). The C-index of the NDSS was 0.838 (95% CI: 0.762-0.914). Additionally, the calibration plots of DSS-B and NDSS had a good coherence between the predictions and actual values in predicting surgical difficulty, as shown in **Figures 5B, C**.

**Table 2:** Patient characteristics and surgical results of laparoscopic liver resection.

Characteristic	High difficult (54)	Low difficult (43)	*P* value
Age, years	60.1 ± 11.4	54.5 ± 13.3	0.030^*^
Gender, male/female	37/17	24/19	0.198
Hypertension, yes/no	21/33	10/33	0.101
Diabetes, yes/no	11/43	6/37	0.409
HOA, yes/no	26/28	14/29	0.121
HOH, yes/no	13/41	3/40	0.048^*^
HOL, yes/no	7/47	0/43	0.016^†*^
Child-Pugh, A/B	53/1	42/1	0.578^$^
Malignant, yes/no	47/7	25/18	0.003^*^
Tumor size, mm	51.2 ± 32.5	42.5 ± 25.3	0.152
DSS-B, L/I/H^a^	5/21/28	21/19/3	<0.001^*^
Pringle, yes/no	49/5	33/10	0.058
Operation time, min	297.1 ± 84.6	152.2 ± 49.7	<0.001^#*^
Bleeding, ml	440.7 ± 368.5	111.6 ± 55.5	<0.001^#*^
Transfusion, yes/no	13/41	0/43	<0.001^†*^
Conversion, yes/no	8/46	0/43	0.008^†*^
POS, days	12.7 ± 6.9	10.0 ± 5.9	0.018^*^
Postoperative morbidity, yes/no	11/43	5/38	0.249
Hemorrhage, yes/no	0/53	1/43	n.s^†^
Bile leakage, yes/no	4/50	0/43	0.127^†^
Ileus, yes/no	1/53	0/43	n.s^†^
Pneumonia, yes/no	2/52	1/42	0.841^$^
Pleural effusion, yes/no	3/51	0/43	0.327^†^
Abdominal infection, yes/no	2/15	0/80	0.501^†^
Abdominal effusion, yes/no	1/53	1/42	0.578^$^
Liver failure, yes/no	1/53	0/43	n.s^†^
Incision infection, yes/no	0/52	2/43	0.194^†^
Postoperative day 1 (POD1)			
ALT, U/L	564.6 ± 553.5	223.7 ± 230.6	<0.001^#*^
AST, U/L	501.5 + 406.1	224.3 + 238.0	<0.001^#*^
TB, umol/L	22.5 ± 12.3	20.2 ± 14.6	0.417
WBC, *10^^^9/L	12.5 ± 4.6	11.7 ± 3.6	0.330
CRP, mg/L	41.2 ± 37.9	45.0 ± 42.0	0.646

HOA, history of abdominal surgery; HOE, history of epigastric surgery; HOH, history of hepatobiliary surgery; HOL, history of liver surgery; POS, postoperative stay; DSS-B, Ban Difficulty Scoring System. n.s, not significant; ALT, alanine aminotransferase; AST, aspartate aminotransferase; TB, total bilirubin; WBC, white blood cell count; CRP, C-reactive protein.

*Statistically significant; ^#^Mann-Whitney test; ^$^Chi-square with Yates’ correction; ^†^Fisher’s exact test.

a L, low (1-5); I, intermediate (6-8); H, high (9-12).

**Table 3:** Surgical outcomes according to the novel difficulty rating system (n=97).

Score	1 (n=1)	2 (n=1)	3 (n=2)	4 (n=3)	5 (n=10)	6 (n=11)	7 (n=9)	8 (n=16)	9 (n=12)	10 (n=14)	11 (n=11)	12 (n=6)	13 (n=1)	*P* value
Operation time, min	55.0	90.0	45.0	95.0 ± 5.0	151.0 ± 21.7	176.8 ± 50.8	237.2 ± 85.9	219.1 ± 96.7	253.8 ± 83.2	266.1 ± 76.5	315.5 ± 57.1	400.8 ± 50.3	330.0	<0.001^*^
Bleeding, ml	50.0	50.0	75.0 ± 35.4	100.0	115.0 ± 53.0	127.3 ± 51.8	283.3 ± 180.3	240.6 ± 215.4	416.7 ± 404.7	357.1 ± 430.9	427.3 ± 462.8	600.0 ± 244.9	800.0	0.030^*^
Conversion, yes/no	0/1	0/1	0/2	1/2	1/9	0/11	1/8	2/14	0/12	3/11	0/11	0/6	0/1	0.592
Transfusion, yes/no	0/1	0/1	0/2	0/3	0/10	0/11	0/9	1/15	3/9	3/11	1/10	5/1	0/1	<0.001^*^
POS, days	4.0	5.0	4.5 ± 2.1	6.7 ± 1.5	9.8 ± 5.0	10.7 ± 7.0	10.1 ± 2.5	11.6 ± 7.7	11.8 ± 7.1	11.6 ± 3.0	12.3 ± 3.6	15.2 ± 14.0	23.0	0.391
ALT, U/L	33.0	1138.0	183.5 ± 177.5	106.7 ± 36.1	220.3 ± 278.3	209.2 ± 180.1	292.9 ± 231.9	392.9 ± 370.7	345.8 ± 380.6	668.1 ± 743.8	414.6 ± 387.5	1002.7 ± 668.8	741.0	0.004^*^
AST, U/L	42	1088	204 199.4	142 ± 90.8	226.6 ± 335.6	207.2 ± 174.9	318.3 ± 247.6	371.5 ± 315.7	338.3 ± 406.4	508.1 ± 412.8	395.1 ± 275.6	876.8 ± 559.1	629	0.012^*^
TB, umol/L	14.8	72.5	11.5 ± 3.3	18.3 ± 4.1	18.2 ± 6.7	25 ± 21.5	17.9 ± 5.1	20.7 ± 11.3	18.2 ± 7.7	20.4 ± 7.8	23.5 ± 19.3	30.9 ± 13.5	21.3	0.026^*^
WBC, *10^^^9/L	12	11.7	11 ± 3.1	10.7 ± 5.5	11.7 ± 3.6	11.9 ± 3.3	11.7 ± 4.2	12.4 ± 5.2	10.1 ± 3.6	13.7 ± 4.5	13.9 ± 4.3	12.1 ± 4.3	10	0.805
CRP, mg/L	1.5	8	24.9 ± 16.1	32.6 ± 24.6	42.5 ± 29.6	54.3 ± 45.9	18.6 ± 10.9	41.9 ± 46.9	47.4 ± 39.2	39.5 ± 43.5	51.4 ± 34.3	75.0 ± 52.0	3.6	0.415
Morbidity, yes/no	0/1	0/1	0/2	0/3	1/9	3/8	1/8	4/12	2/10	1/13	1/10	2/4	1/0	0.525

POS, postoperative stay; ALT, alanine aminotransferase; AST, aspartate aminotransferase; TB, total bilirubin; WBC, white blood cell count; CRP, C-reactive protein.

*Statistically significant.

### Subgroup analysis

**Figure 6:**
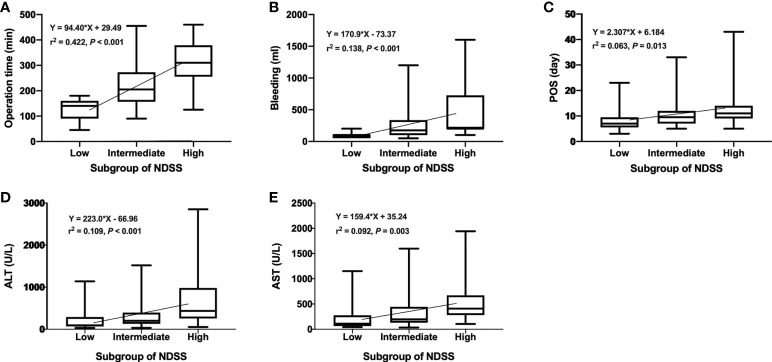
Operation time **(A)**, bleeding **(B)**, POS **(C)**, ALT **(D)**, and AST **(E)** according to subgroups.

We compared the intraoperative outcomes among cases classified as low (NDSS 1-5), intermediate (NDSS 6-9), high (NDSS 10-14) difficulty. The results showed that the operation time (*P* < 0.001), blood loss (*P* = 0.001), transfusion (*P* = 0.008), POS (*P* = 0.041), ALT (*P* = 0.002) and AST (*P* = 0.006) were significantly different among these subgroups (**Table 4**). And its correlation was shown in **Figure 6**.

**Table 4:** Surgical outcomes according to the novel difficulty scoring system (n=97).

Subgroup	Low (n=17)	Intermediate (n=48)	High (n=32)	*P* value
Operation time, min	119.4 ± 44.8	221.5 ± 84.8	310.3 ± 80.0	<0.001^*^
Bleeding, ml	100.0 ± 46.8	266.7 ± 264.2	440.6 ± 411.0	0.001^*^
Conversion, yes/no	2/15	3/45	3/29	0.746
Transfusion, yes/no	0/17	4/44	9/23	0.008^*^
POS, days	8.0 ± 4.5	11.1 ± 6.5	12.8 ± 6.7	0.041^*^
ALT, U/L	238.9 ± 320.6	320.3 ± 314.3	646.0 ± 629.9	0.002^*^
AST, U/L	248.8 ± 340.9	315.6 ± 302.3	542.2 ± 420.1	0.006^*^
TB, umol/L	20.4 ± 14.6	20.5 ± 12.8	23.5 ± 13.8	0.599
WBC, *10^^^9/L	11.5 ± 3.5	11.6 ± 4.2	13.4 ± 4.3	0.456
CRP, mg/L	34.2 ± 27.4	41.8 ± 40.9	49.1 ± 43.0	0.444
Morbidity, yes/no	1/16	10/38	5/27	0.356

POS, postoperative stay; ALT, alanine aminotransferase; AST, aspartate aminotransferase; TB, total bilirubin; WBC, white blood cell count; CRP, C-reactive protein.

Subgroup: Low, score = 1-5; Intermediate, score = 6-9; High, score = 10-14.

^*^Statistically significant.

### Development and validation the nomogram of adverse event

**Figure 7:**
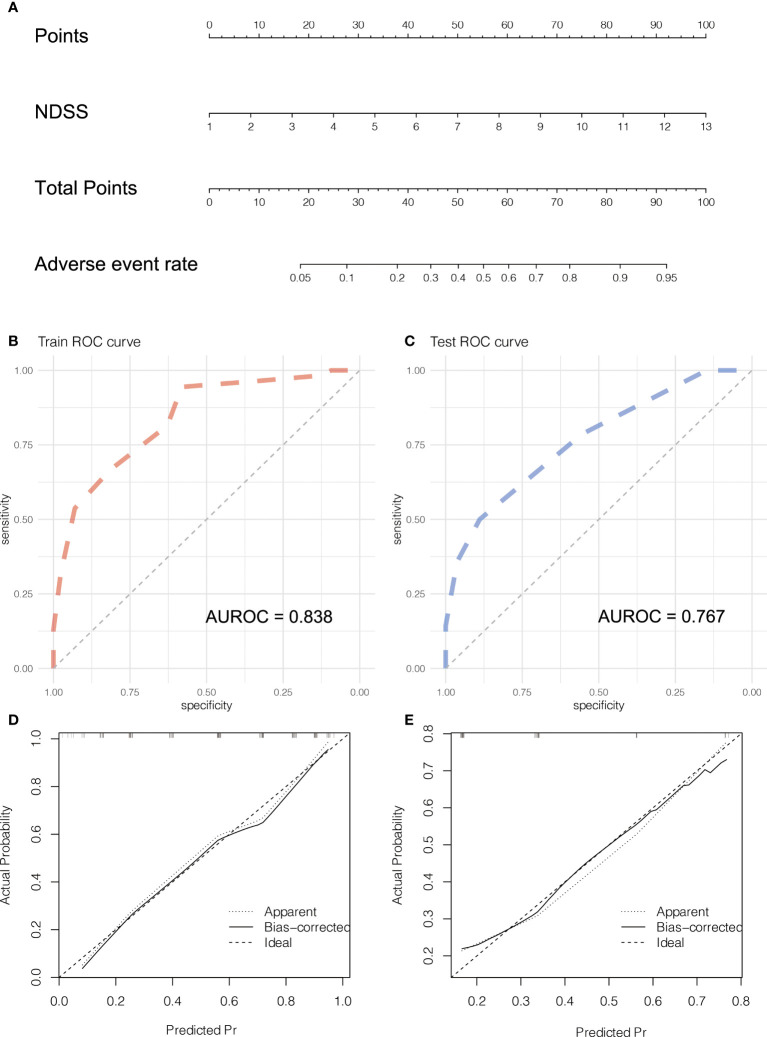
Development and validation the Nomogram of adverse event. The nomogram **(A)** of adverse event. The ROC curve of the nomogram in training **(B)** and testing **(C)** cohort. The calibration plots of the nomogram in training **(D)** and testing **(E)** cohorts.

The NDSS were incorporated into the nomograms (**Figure 7A**). In the training cohort, the AUROC of the nomograms was 0.833 (**Figure 7B**). To validate the nomogram, 41 patients (testing cohort) who performed LLR were selected from Wujin Hospital Affiliated with Jiangsu University. The characteristics between training and testing cohort were shown in **Table 5**. In the testing cohort, the AUROC of the nomogram for predicting the adverse event (the degree of surgical difficulty) was 0.767 (**Figure 7C**). The calibration plots of the nomogram had a good coherence between the predictions and actual values in the probability of adverse event in both training and testing cohorts, as shown in **Figures 7D–E**.

**Table 5:** Patient characteristics and surgical outcomes of LLR between Training and Testing cohorts.

Characteristic	Training group (97)	Testing group (41)	*P* value
Age, years	57.7 ± 12.6	58.9 ± 12.8	0.610
Gender, male/female	61/36	24/17	0.631
Hypertension, yes/no	31/66	16/25	0.424
Diabetes, yes/no	17/80	10/71	0.089
HOH, yes/no	16/81	2/39	0.115
POS, days	11.2 ± 6.4	9.4 ± 3.7	0.221^#^
Child-Pugh, A/B	95/2	38/3	0.312^$^
Tumor size, mm	47.3 ± 29.7	40.0 ± 24.2	0.531
NDSS, L/I/H^a^	26/30/31	2/33/6	0.765^&^
Pringle, yes/no	82/15	33/8	0.560
Operation time, min	232.9 ± 101.3	210.0 ± 77.89	0.199
Bleeding, ml	294.8 ± 321.4	206.2 ± 371.8	0.160
Transfusion, yes/no	13/84	2/39	0.242^$^
Conversion, yes/no	8/73	1/40	0.264^$^
Malignant, yes/no	72/25	21/20	0.008^*^
Postoperative morbidity	16/81	2/39	0.115^$^
Hemorrhage, yes/no	1/96	0/41	n.s^†^
Bile leakage, yes/no	4/93	1/40	0.989^$^
Ileus, yes/no	1/96	0/41	n.s^†^
Pneumonia, yes/no	3/94	0/41	0.555^†^
Pleural effusion, yes/no	3/94	2/39	0.989^$^
Abdominal infection, yes/no	2/95	0/41	n.s^†^
Liver failure, yes/no	1/96	0/41	n.s^†^
Incision infection, yes/no	2/95	0/41	n.s^†^
Postoperative day 1 (POD1)			
ALT, U/L	413.5 ± 470.5	215.7 ± 338.0	<0.001^#*^
AST, U/L	378.6 + 367.4	195.0 + 250.2	<0.001^#*^
TB, umol/L	21.5 ± 13.4	21.7 ± 7.5	0.106^#^
WBC, *10^9/L	12.1 ± 4.2	10.8 ± 3.5	0.065
CRP, mg/L	42.9 ± 39.6	25.7 ± 10.0	0.308^#^

HOH, history of hepatobiliary surgery; POS, postoperative stay; NDSS, novel difficulty scoring system; n.s, not significant; ALT, alanine aminotransferase; AST, aspartate aminotransferase; TB, total bilirubin; WBC, white blood cell count; CRP, C-reactive protein.

^*^Statistically significant; ^#^Mann-Whitney test; ^$^Chi-square with Yates’ correction; ^&^Chi-square test for trend; ^†^Fisher’s exact test.

a L, low (1-5); I, intermediate (6-9); H, high (10-14).

## Discussion

LLR has rapidly become widespread all over the world (11). In recent years, more and more surgical centres have included laparoscopic hepatectomy in the routine treatment of liver tumors, and the proportion is gradually increasing, and is up to 30.8% of liver resection (LR) (3). At the European Guidelines Meeting for Laparoscopic Liver Surgery, it was noted that LLR was a complex surgical skill that must be mastered in a progressive manner (12). The conventional advice is to start with a small or left lateral lobectomy and then perform a major resection as the experience increases. In addition, this simplification overlooked factors that affect laparoscopic liver resection difficulties difficulty, such as the relationship between neoplasms and large vessels and the history of liver resection. Hence a simple, objective, and robust preoperative difficulty scoring system could help surgeons master the procedure step by step.

For the past few years, a difficulty grading system of LLR has been proposed by experts (7, 13–17). Ban et al. analyzed clinical data and difficulty index of 30 patients, screened out 5 independent risk factors affecting LLR difficulty, and established the DSS-B using a linear regression model (7). The impact of different types of laparoscopic surgery was not considered in DSS-B, such as total laparoscopic hepatectomy, hand-assisted laparoscopic hepatectomy, on the difficulty of LLR, making the difficulty score incomplete. Therefore, some scholars improved DSS-B and launched an upgraded version of the IWATE Criteria for difficulty scoring (13). Different from DSS-B, Hasegawa et al. used operation time as the indicator of surgical difficulty, evaluated the influence of preoperative factors on operation time through multiple linear regression analysis, and included BMI as a difficulty scoring factor for the first time (14). In 2018, Kawaguchi et al. developed a difficulty scoring system based on the extent of resection (DSS-ER) (15). For the first time, operation time, blood loss and conversion rate were used as difficulty criteria, and the median was used as the cutoff value. However, this scoring system was based on the intraoperative results and ignored the preoperative and postoperative factors, and the verification was carried out by postoperative results, which might have a certain bias. In addition, it only considered the type of resection and ignored the influence of factors such as the general state of the patients and tumors on the operation, so its accuracy might be affected. In the same year, Halls et al. reported on a difficulty scoring system based on data from seven centres (16). For the first time, previous open liver surgery history and preoperative neoadjuvant chemotherapy were factored into the scoring system. However, the definition of preoperative neoadjuvant chemotherapy in this study was very vague, and there was a lack of data about the time and cycle of neoadjuvant chemotherapy, so the actual prediction results might be biased to some extent. Subsequently, Tong et al. proposed Sir Run Run Shaw Hospital (SRRSH) risk models based on conversion and complication. For the first time, this scoring system included the American society of anesthesiologists (ASA), ALT, cirrhosis and other indicators that reflect the general situation of patients and was a prediction model for the feasibility and safety of LLR. However, most of the cases in this study were small-scale hepatectomy, which might lead to selection bias. Therefore, more cases were needed to prove the application of SRRSH score in large-scale hepatectomy. The difficulty scoring system reported previously did not contain all the factors affecting surgery, which affects their accuracy. In addition to the above factors, Guilbaud et al. reported that an estimated parenchymal transection surface area ≥ 100 cm^2^ was a relevant indicator of surgical difficulty and postoperative complications in LLR (18).

In the present study, the high difficulty outcome events were identified as blood loss > 400 ml, and operation time > 240 min or conversions. The measures of surgical difficulty were similar to DSS-ER (15). But the specific reference values were different. This difference may have to do with the different measures used by different centres. Through correlation analysis, age, history of hepatobiliary surgery (HOH), history of liver surgery (HOL), and malignant and DSS-B were closely related to surgical difficulty. Based on clinical experience and the results of other centres, age was not a direct factor affecting the difficulty of LLR. The treatment of malignant tumors requires radical excision, while benign tumors can be excised close to the tumor without worrying about positive margins. Moreover, malignant tumors are often accompanied by changes in liver texture, such as hepatocellular carcinoma, which is often accompanied by hepatitis, hepatic fatty degeneration, or alcoholic liver. These changes were not sufficient to cause significant liver function damage and affected Child-Pugh score but increased operation time during surgery. There was an overlap between patients with HOH and patients with HOL. So, we selected HOH, malignant and DSS-B to build the novel difficulty scoring system. The novel system was an improvement of the classical model DSS-B. And the novel system was better than DSS-B according to the ROC curve.

Although there was no correlation between surgical difficulty and postoperative complications in this study, some studies have shown that highly difficult LLR might increase the incidence of postoperative complications (9, 15). High difficult LLR may lead to longer operation time and more blood loss, resulting in a higher incidence of postoperative complications. In these patients, laparoscopic hepatectomy should be carefully determined and recommended only in high-volume centres with an experienced team. Thus, more difficult cases would be taken over by more qualified surgeons (19). In addition to postoperative complications, the relationship between the difficulty grade of laparoscopic liver resection for malignant tumor and the long-term outcomes is of great concern to scientists (20). A growing body of evidence indicates that postoperative complications, which in our series increased along with LLR difficulty, trigger the systemic proinflammatory cascade through the release of cytokines such as IL-1b, IL-6, TNF-a, oxidative stress, and immunosuppression and consequently promote tumorigenesis and metastatic spread (21–23). Postoperative complications have a negative impact on overall survival and disease-free survival in all types of malignancies (24, 25). In addition, failure or delayed administration of adjuvant therapy due to postoperative complications may increase tumor recurrence and affect survival. In addition, with the increase of LLR difficulty, the significant increase in intraoperative blood loss and transfusion ratio was also a risk factor for poor short-term and long-term prognosis of various malignant tumors (26).

In addition to liver tumors, laparoscopic liver resection can also be used for intrahepatic duct (IHD) stones. Kim et al. developed a modified difficulty scoring system for IHD stones (10). The technical requirements of laparoscopic hepatectomy for IHD stones appear to be higher than for tumors, as the liver inflammation associated with IHD stones can lead to perihepatic adhesion and anatomic distortion. In addition, additional choledochoscopy of the remaining biliary tract is often required intraoperatively, which increases surgical complexity and prolongs surgical time. Therefore, under the same circumstances, laparoscopic liver resection for IHD stones is more difficult than liver tumors, and the two are not applicable to the same difficulty scoring system.

The use of surgical robots in liver surgery is growing almost daily. The robot offers a three-dimensional image with instruments of seven degrees of freedom (27). Compared with laparoscopic surgery, the main advantages of the robot are its ergonomic design, superior flexibility and visualization, which may better simulate open surgery and solve some operational difficulties in laparoscopic hepatectomy. However, the robotic hepatectomy is still a cutting-edge technology for liver surgeons, which requires a certain learning process. It is not clear whether the previous difficulty scoring system is suitable for robotic hepatectomy. Therefore, Chong et al. validated the DSS-B in robotic hepatectomy and to compare the outcomes of robotic hepatectomy and conventional laparoscopic hepatectomy among different difficulty levels (28). The results suggest that the benefits of the robotic platform may be minimal in moderate-to-low difficulty hepatectomy. However, robotic approaches make high difficulty liver resection more minimally invasive.

There are multiple advantages to the present study. Firstly, history of previous abdominal surgery was included in the evaluation system of surgical difficulty of LLR. Additionally, this study is the first to develop a nomogram related to the difficulty of laparoscopic hepatectomy. However, the limitations of this study include its retrospective nature, and the lack of subgroup analysis of malignancies. The liver texture of hepatocellular carcinoma is different from that of metastatic liver tumors. In addition, some studies have shown that preoperative neoadjuvant chemotherapy also has a certain impact on liver resection for metastatic liver cancer, which was not reflected in this study (20, 29).

## Conclusion

In conclusion, we improved the DSS-B and proposed a new classification system of LLRs according to their surgical difficulty. This system provides 3 difficult levels of LLRs: low difficulty, intermediate difficulty, and high difficulty. This classification could more accurately reflect the difficulty of surgery and help liver surgeons to make the surgical plan and ensure the safety of the operation. As surgeons gain experience, they can choose appropriate patients and gradually progress from a low level of expertise to an advanced level of expertise.

## Data availability statement

The raw data supporting the conclusions of this article will be made available by the authors, without undue reservation.

## Ethics statement

The studies involving human participants were reviewed and approved by the Affiliated Hospital of Jiangnan University ethics committee. Written informed consent for participation was not required for this study in accordance with the national legislation and the institutional requirements.

## Author contributions

CX and MZ: Study design, Data collection, Writing. TJ and YT: Study design, Data collection, Revision. LZ, ZY and ZZ: Study design, Data analysis. LX and ZL: Study design and Data analysis. WD, XX and WX: Study design, Data collection, Data analysis, Writing, Revision. All authors contributed to the article and approved the submitted version.

## Funding

This work was supported by the Changzhou Sci&Tech Program (CJ20210013, CJ20220008), Young Talent Development Plan of Changzhou Health Commission (CZQM2020118, CZQM2021028), the Development Foundation of Affiliated Hospital of Xuzhou Medical University (XYFY2020016), Medical Research Project of Jiangsu Health Commission (No. Z2019027), Changzhou High-Level Medical Talents Training Project.

## Acknowledgments

The authors acknowledge for colleagues in the Department of Hepatobiliary Surgery, Affiliated Hospital of Jiangnan University. Thanks for the support of Changzhou High-Level Medical Talents Training Project.

## Conflict of interest

The authors declare that the research was conducted in the absence of any commercial or financial relationships that could be construed as a potential conflict of interest.

## Publisher’s note

All claims expressed in this article are solely those of the authors and do not necessarily represent those of their affiliated organizations, or those of the publisher, the editors and the reviewers. Any product that may be evaluated in this article, or claim that may be made by its manufacturer, is not guaranteed or endorsed by the publisher.
